# Off-label use of orthopedical trauma implants in a low-income country

**DOI:** 10.1007/s00264-021-04990-x

**Published:** 2021-02-26

**Authors:** F. Wichlas, V. Hofmann, G. Strada, M. Moursy, C. Deininger

**Affiliations:** 1 University Clinic for Orthopedics and Traumatology, Paracelsus Medical University, Müllner Hauptstrasse 48, 5020 Salzburg, Austria; 2 No Limit Surgery, Ernest-Thun-Strasse 6, 5020 Salzburg, Austria; 3 Emergency NGO, Milan, Italy

**Keywords:** Off-label treatment, NGO surgery, Austere environment, Complex injuries, LIC, K-wires, External fixators

## Abstract

**Purpose:**

Lack of resources, severe injuries, and logistical flaws force surgeons in low-income countries (LIC) to improvise during surgery and use implants “off-label.” These off-label treatments are specific for the work of trauma surgeons in non-governmental (NGO) hospitals in LIC. The aim of this study is to show the need of off-label surgery in an environment of low resources by means of typical examples.

**Methods:**

Off-label treated fractures, the implant used instead, and the reason for off-label treatment were investigated in 367 injuries over a three month period in an NGO hospital in Sierra Leone.

**Results:**

Twenty-seven fractures were treated off-label with mostly K-wires (88.89%) and external fixators (51.85%). Three reasons for off-label use could be defined: no suitable implants (*N* = 14), the condition of soft tissues that did not allow internal osteosyntheses (*N* = 10), and implants not ready for surgery due to logistic flaws (*N* = 3). The implants needed were mostly locking plates.

**Conclusion:**

Surgeons in similar settings must use K-wires and external fixators to treat complex fractures. Using implants off-label can help surgeons to treat fractures otherwise left untreated.

## Purpose

Surgery in low-income countries (LIC) has evolved differently than in high-income countries (HIC) and has another standard of care [1]. Limited resources combined with high patient inflow result in a mismatch of the hospitals’ capacity and its workload [2, 3]. Implants are often not available or unaffordable, medical personnel training varies widely, and severe injuries are often [4]. This leads to fractures treated conservatively, or not at all, that might have needed surgical treatment to restore adequate function. For expatriate surgeons in non-governmental (NGO) hospitals, this situation is unfamiliar because in HIC implants are always available and the personnel is usually trained. But as some LIC develop rapidly and NGO hospitals improve, especially with regard to the equipment of the operating theatres (OT), a limited number of implants become available, such as external fixators or SIGN nails [5–7]. So-called HIC standards of care are hard to hold and the surgeons must treat complex injuries with these available implants at any time. The resulting surgery is neither classic LIC surgery nor HIC surgery but a mix of both. Improvisation is a motor to this development of surgery in austere and war regions.

This development often begins with “off-label treatment” of fractures caused by the act of necessity. An airway tube filled with bone cement used as an external fixator for mandibula fractures, known as “Joe Hall Morris fixation,” is a good example for off-label use [8]. Special implants for some complex injuries might not be available. Serious involvement of soft tissues makes the standard osteosynthesis impossible and casting would only postpone the problem to a later moment. The surgeon might be confronted with situations resulting from procedural mistakes where he has no choice but to work with what is available at the moment and use implants and techniques in a way they were not meant to be, in an “off-label” way. Off-label treatments are specific for the work of trauma surgeons in NGO hospitals in LIC.

The goal of this work was to identify the off-label treatments typical for an NGO hospital in a LIC. The off-label osteosyntheses and the reasons for them were evaluated.

The results of this study could provide alternative solutions for surgeons in similar settings and help them to create solutions on their own.

## Methods

The patients analyzed were admitted to an NGO hospital in Freetown, Sierra Leone, Africa. It had 85 beds, eight intensive care beds without ventilator, three OTs, an outpatient department (OPD), a room for casting/splinting, and one for physiotherapy. The OTs were new (1 year) and ensured a high standard of hygiene compared to other LIC. A C-arm and an electric power drill were available.

The orthopaedic implants available were small and large external fixators (Hoffmann II external fixator system and Hoffmann II compact, Stryker Trauma AG, Selzbach, Switzerland, and AO external steel fixator, Depuy Synthes, Oberdorf, Switzerland), intramedullary nails (SIGN Fracture Care International, Richland, WA, USA), K-wires (steel, 1.2 to 4 millimeters), and non-locking small and large fragment low contact steel plates (Braun Aesculap, Tuttlingen, Germany). The admission criteria to the hospital were trauma victims, patients requiring general surgery, and paediatric patients.

### Epidemiology

From the tenth of October 2015 to the eight of January 2016 (3 months), data from 282 patients (205 male, 77 female; 211 adults, 71 children) with 367 injuries were recorded prospectively; 273 had 349 fractures (184 left, 150 right side) and nine had none. More than one bone was fractured in 63 patients (22.34%). The causes of injury were road traffic accidents (RTA, *N* = 215, 76.24%), falls (*N* = 59, 20.57%), falls from height (*N* = 6, 2.13%), and stab wounds (*N* = 3, 1.06%). During 64 days, 263 surgical procedures  were performed and 185 patients were treated with one or more osteosyntheses. The whole dataset of this population has been published before [9].

We identified fractures that were treated off-label. Off-label treatment of fractures, in the context of this study, meant using osteosyntheses differently than they would be used “as textbook standard” in HIC or differently than they were planned. We determined the implant used instead, investigated the reason for the off-label use, and if the fractures were open or closed. The anatomical region of the fracture was noted.

Differences for categorical variables were assessed with the chi-square test. Differences were considered statistically significant if the null hypothesis could be rejected with >95% confidence (*P* < 0.05).

## Results

In 25 patients (20 males; 5 females), 27 fractures (7.36% of 367 injuries) were treated off-label. The mean age was 31.35 years (range from 6 to 63 years).

Table 1 shows the anatomical regions, the implant used instead, the reason for the off-label use, and whether the fracture was open or closed.

**Table 1:** The anatomical AO classified regions of the fractures (number, percentage), the number of patients, the reason for the off-label use, the implant used instead, and whether the fracture was open or closed

Region (*N* / %)	P	Impl	STD	NR	K-W	Ex Fix	Screw	Nail	Cast	Op	Clo
Femoral (1 / 3.70%)	1	1	0	0	0	1	1	0	0	0	1
Distal, AO 33 (1)		1	0	0	0	1	1	0	0	0	1
Tibial (17 / 63.00%)	16	9	5	3	17	9	2	1	6	9	8
Proximal, AO 41 (2)		1	1	0	2	1	1	0	0	0	2
Shaft, AO 42 (6)		4	0	2	6	3	0	0	3	3	3
Distal, AO 43 (4)		3	1	0	4	2	0	1	1	3	1
Malleolar, AO 44 (5)		1	3	1	5	3	1	0	2	3	2
Foot (1 / 3.70%)	1	0	1	0	1	1	0	0	0	1	0
Crushed foot (1)		0	1	0	1	1	0	0	0	1	0
Humeral (1 / 3.70%)	1	1	0	0	0	0	0	0	1	0	1
Shaft, AO 12 (1)		1	0	0	0	0	0	0	1	0	1
Forearm (4 / 14.80%)	4	1	3	0	4	1	0	0	0	2	2
Shaft, AO 22 (4)		1	3	0	4	1	0	0	0	2	2
Hand (1/ 3.70%)	1	0	1	0	1	1	0	0	0	1	0
Crushed hand (1)		0	1	0	1	1	0	0	0	1	0
Maxilla (2 / 7.41%)	2	2	0	0	1	1	1	0	0	1	1
Total		14	10	3	24	14	4	1	7		

*P*, patients; *%*, percent; *Impl*, no implant; *STD*, soft tissue defect; *NR*, implant not ready; *K-W*, K-wires; *Ex Fix*, external fixator; *Op*, open fracture; *Clo*, closed fracture

The implants most used for off-label surgery were in 88.89% K-wires and in 51.85% external fixators.

Three reasons for off-label treatment of fractures were identified:

- No implant
- Condition of the soft tissues
- Implant not ready

“No implant” resulted from the limited resources in the hospital. Specific “modern” implants, especially locking plates, were not available.

“Condition of soft tissues” resulted from the serious damage to the soft tissues due to the injury. The damage did not allow conventional internal osteosyntheses.

“Implant not ready” resulted from implants not sterilized on time or that ran out due to supply problems.

Table 2 shows the amount of open and closed fractures in these 3 groups.

**Table 2:** The amount of open and closed fractures in the 3 groups

	Fractures	Open	Close
No implant	14	4	10
Condition of soft tissues	10	8	2
Implant not ready	3	2	1

**Fig. 1:**
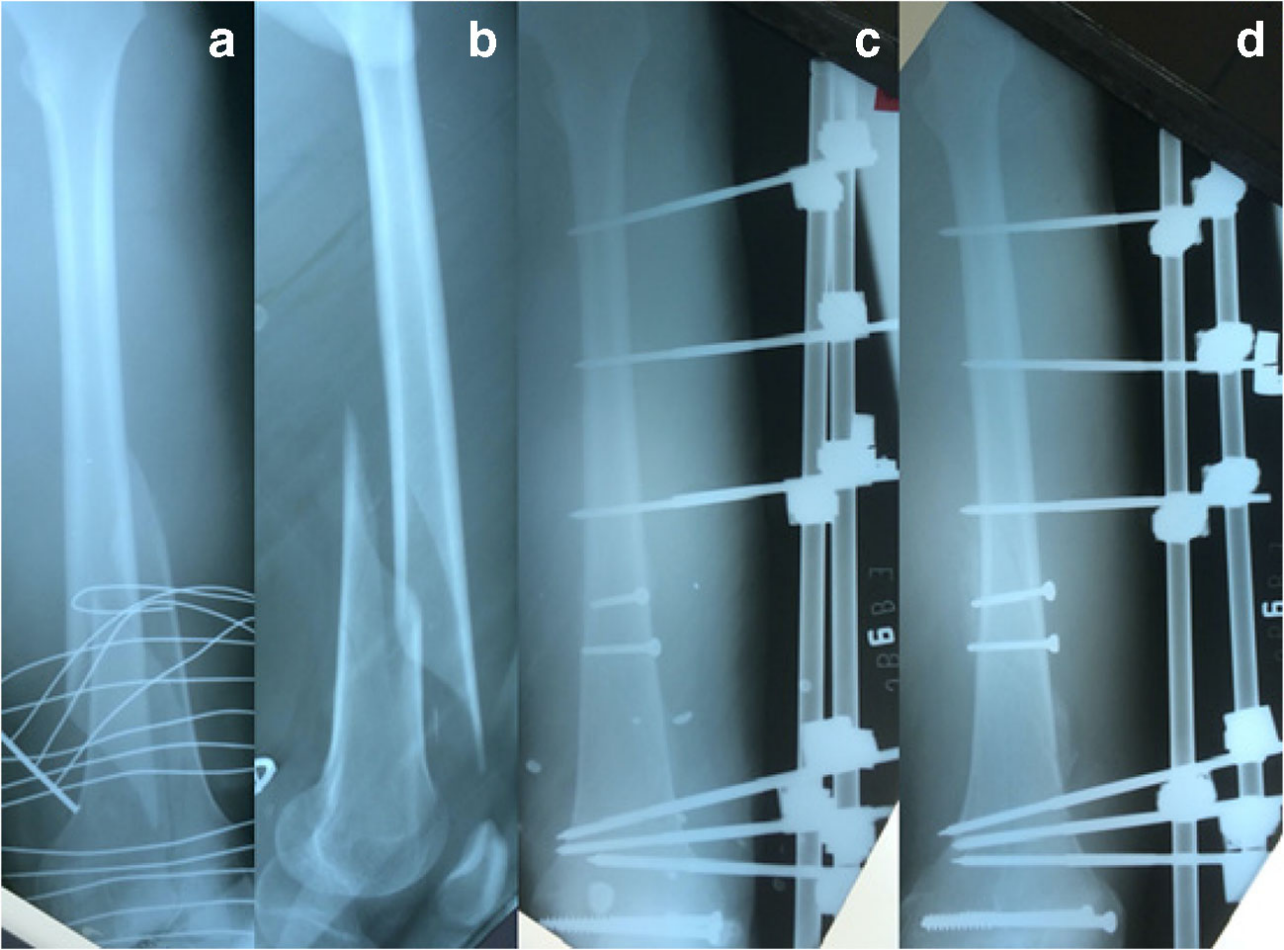
No implant. A 35-year-old male sustained a road traffic accident with I ° closed femoral fracture AO 33 A1, left side. The fracture extends into the articular surface. Pre-operative anteroposterior and lateral X-ray of the fractured femur (**a** and **b**). In HIC, a locking plate would be the implant of choice. We fixed the articular surface and the shaft component with two screws each (4.5 mm cortex screws and 6.5 mm cancellous screws, steel) and the locking plate was replaced by an external fixator. Anteroposterior and lateral X-ray 8 weeks postoperatively of the left femur showing the osteosynthesis in place and callus formation on the fracture site (**c** and **d**). Postoperative treatment was partial weight bearing and no limitation for knee and hip

The main reason for off-label use was that no implants (3.81% of all 367 fractures) were available. In 13 out of 14 fractures, locking plates were missing implant (Fig. 1). In this group, there were more closed fractures (*N* = 10) than open ones (*N* = 4), when compared to the rest (*P* = 0.012). The implants used were K-wires in 3 fractures, external fixators and K-wires in four, K-wires and screws in three, and K-wires and a nail in one. The external fixator was used once in combination with screw and once alone. One fracture, in the distal humeral shaft, was casted. The fracture region in long bone shafts, four tibiae and one humerus, was mainly close to the joint and therefore not amendable for the SIGN nail. Once no standard of care implant could be proposed for a III°B open comminuted distal tibial shaft fracture in a child.

**Fig. 2:**
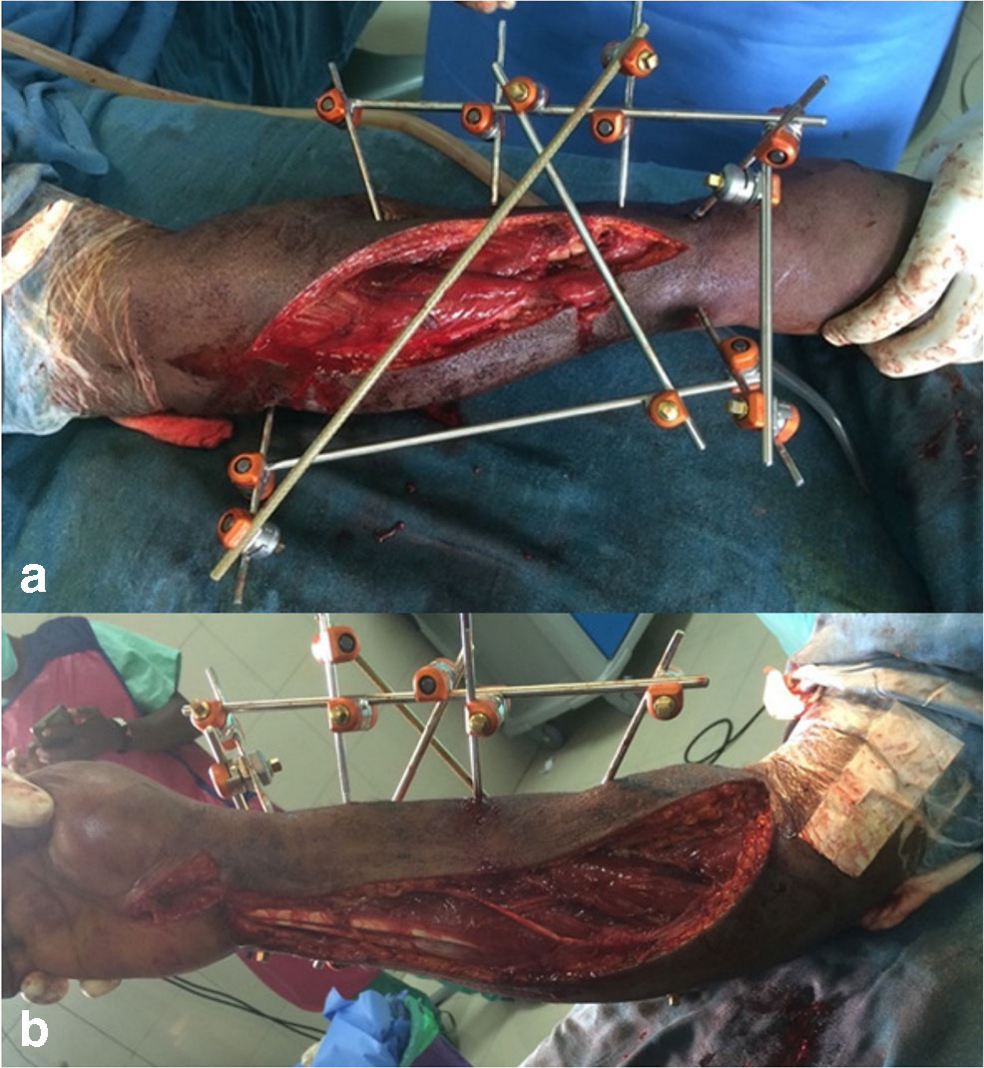
Condition of soft tissue. Severe forearm injury, treated off -label because of III°B open soft tissue injury. This 41-year-old male patient got in a traffic road accident and injured his right forearm (Fracture AO 2R2A2/2U2C2, G III° B open, compartment syndrome). The patient claimed that a car rolled over his forearm. The initial treatment was external fixation and dorsopalmar dermatofasciotomy. The fixation of the ulna was later aligned with an intramedullary K-wire, the palmar wound closed, and the dorsal one was mesh grafted. Although the external frame fixation might not be very common, external fixation is the treatment of choice for open fractures. To achieve a better alignment and increase the chance for orthograde healing, we decided to add an intramedullary K-wire for the ulna. Intramedullary K-wires are unusual or off-label for forearm fractures in adults. Posterior (**a**) and anterior (**b**) clinical picture after dorsopalmar dermatofasciotomy and application of an external fixator

**Fig. 3:**
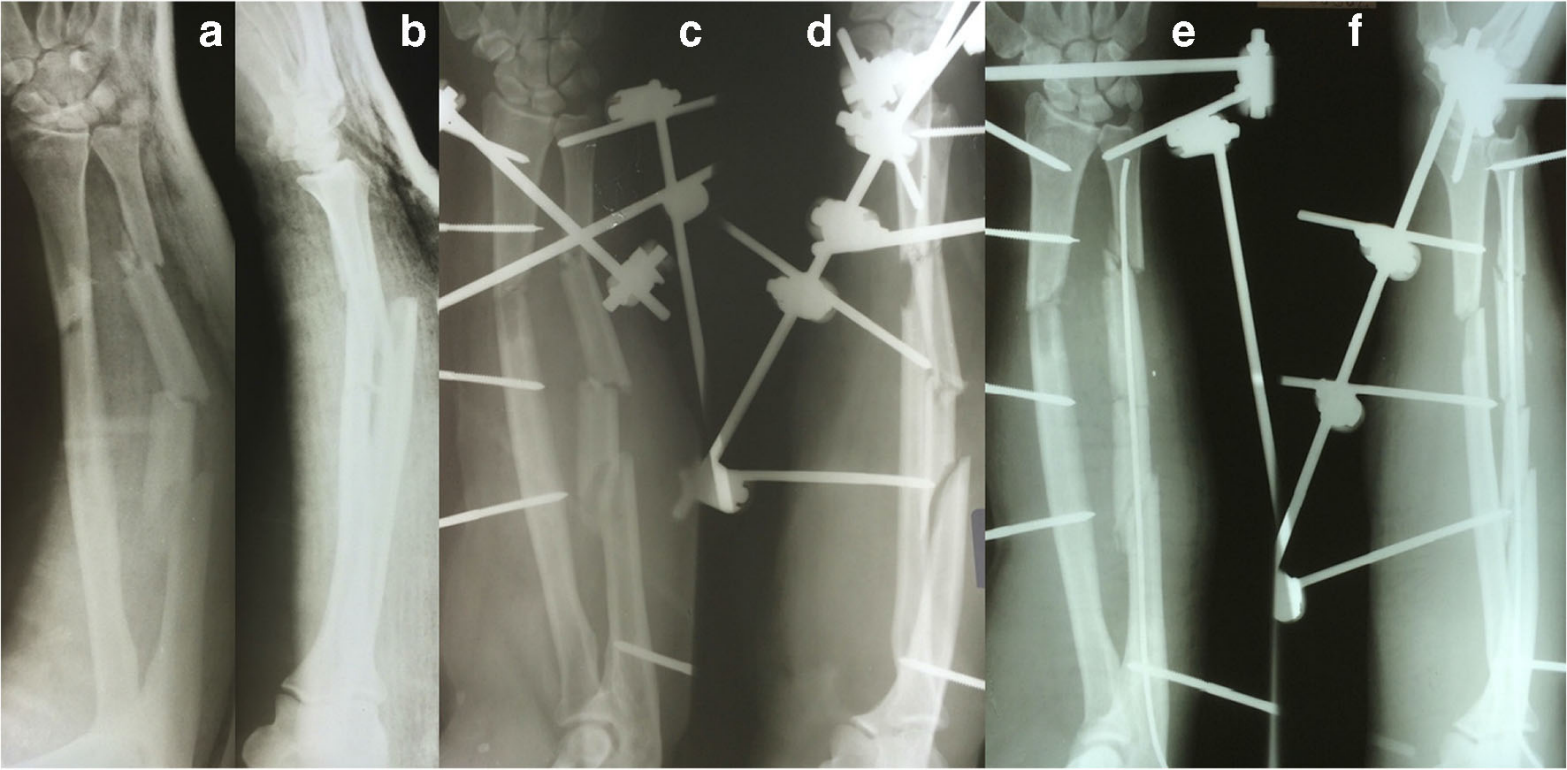
Anterior and lateral preoperative X-ray of the fractured forearm (**a** and **b**), III°B open of the patient mentioned in Fig. 2. Post-operative anteroposterior and lateral X-ray of the forearm showing the external fixator in place (**c** and **d**). Post-operative anteroposterior and lateral X-ray of the forearm showing the external fixator and an intramedullary K-wire to align the fracture (**e** and **f**). The K-wire was inserted at the last surgery before the skin was closed

The condition of the soft tissues (2.72% of all 367 fractures), III° open or III° closed (*N* = 10), was the second reason for off-label use (*N* = 10). In this group, there were more open fractures (*N* = 8) than closed ones (*N* = 2), when compared to the rest (*P* = 0.025). In 4 fractures, the soft tissue defect needed flap coverage. All these cases were treated with K-wires, seven times in combination with an external fixator (Figs. 2 and 3). The standard of care implant was six times a locking plate. Four times no standard of care treatment could be proposed for two malleolar fractures, one crushed foot, and one distal tibial fracture.

**Fig. 4:**
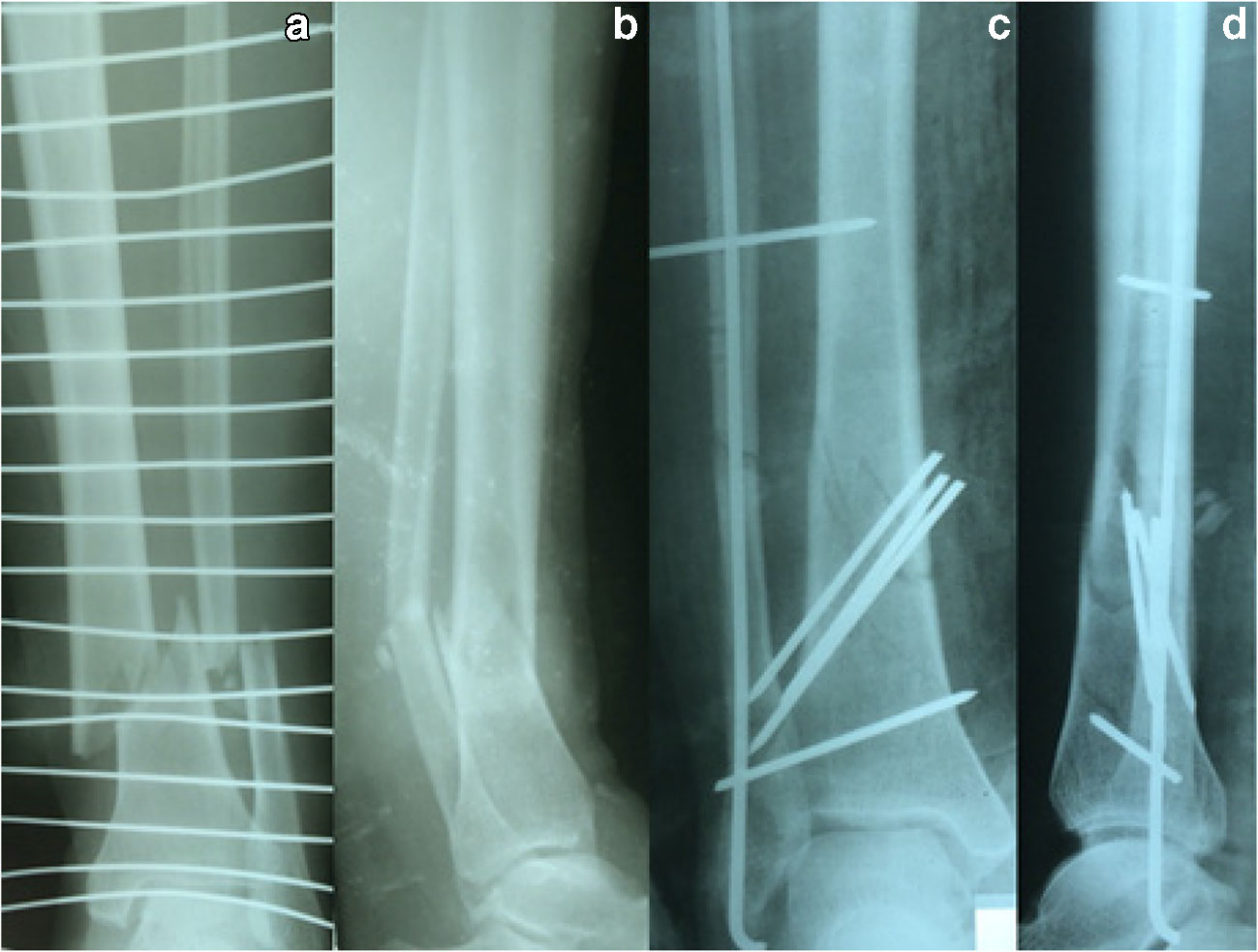
Implant not ready. A 34-year-old female patient sustained a III°B open tibial fracture AO 42 A2.3 by a road traffic accident. Anteroposterior and lateral X-ray of the fractured lower leg, III°B open (**a** and **b**). The patient was planned for external fixation as an emergency procedure. At the time of operation, no external fixator was ready in the OT. After debridement, we decided to fix the fracture with K-wires additionally to a cast. The K-wires should provide an anatomic reduction that the cast alone could not. The three intrafocal K-wires should hold the reduction of the tibia, the intramedullary K-wire align the fibula, and the two fibulotibial K-wires act as a frame fixation (**c** and **d**). As the K-wires would become loose and migrate, their intention is to hold the anatomic reduction as long as possible

In three cases, the implant was available but not ready (0.82% of all 367 fractures). All were treated with K-wires, once in combination with an external fixator (Fig. 4). Twice an external fixator was not ready for urgent surgery, once plates and screws (3.5 mm) for planned surgery.

## Discussion

K-wires (88.89%) and external fixators (51.85%) were used the most for off-label surgery in this NGO hospital.

K-wires are universally applicable in nearly every bone, in most paediatric fractures, and are comparably cheap. In this hospital, their availability had practically no limit. However, they are biomechanically unstable and usually require an additional external fixation, usually a plaster of Paris or a cast. Furthermore, these implants need a power drive to be used and, although not absolutely necessary, an image intensifier. In LIC, K-wires have been showed to be extremely valuable for treatment of hand, foot, and paediatric fractures [9].

The external fixator is the Swiss army knife of NGO surgery in LIC; the use of it has practicably no limits in application and materials used [8, 10–16]. External fixators seem to be made for austere conditions. Even when electric power drills are not available, practicable external fixator systems exist. The fact that 35.24% of the fractures are open fractures [9] makes these implants indispensable. Additionally, external fixators are reusable and the angle stability permits treatment even in weak bone. However, its availability may be limited due to this reusability. At one point in Sierra Leone, almost all 70 external fixators were in use at the same time and none was left for further treatment. In this setting, the use of external fixators requires some planning and in some fractures, the fixators need to be removed and replaced by a cast before fracture healing to use it for the next fracture.

No implants and the condition of the soft tissues were the main reason for off-label surgery (7.36% of all injuries) in this NGO hospital.

The lack of implants was mostly relevant for closed fractures. Contrary to open fractures, closed ones can be treated by any osteosynthesis without increasing the risk for infection [17]. Locking plates were the implants not available and, in our opinion, mostly needed, especially for articular fractures or shaft fractures in close proximity to the joint.

The lack of implants can be overcome by acquisition of implants or improvisation such as off-label use. Although NGOs have become very efficient in the acquisition of implants [18], the availability of implants changes from one organization to another, and from country to country depending on NGO’s internal decisions [19]. While some hospitals are well equipped, others have a very basic set of implants. Nevertheless, even the well-equipped ones are usually still far from western standards and the new equipment needs to be serviced and refilled [20]. All these factors lead to limited access of implants and the necessity to develop exit strategies and improvise. This process is, nevertheless, effortful in an exhausting setting and requires a lot of expertise of the surgeon in charge. Furthermore, as European surgeons, we are used to get every instrument we deem necessary almost immediately and in impeccable condition. During this study, our own inflexibility led us to early saturation and resignation. Hence, our limit of improvisation was reached at 3.81% of all injuries during this study due to lack of implants. As the availability of implants is usually determined, we think that surgical off-label skills and improvisation are the mainstay for the solution of this problem.

Severe soft tissue defects were another reason for off-label treatment. In these fractures, the risk for infection is the highest [21]. Not many implants are left for this situation but external fixators or, as the volume of implant is a key factor for infection, K-wires [17]. In HIC countries, these fractures are usually treated with primary external fixation and secondary definitive surgery including internal osteosynthesis and, if necessary, plastic-surgical reconstruction requiring an interdisciplinary team of surgeons. In LIC, the surgeon’s own expertise is crucial for the treatment of these cases, as he needs experience in all disciplines necessary at once, orthopaedic trauma, vascular, and plastic surgery. Alternatively, to these skills, the surgeon should be familiar with alternative or past techniques well known and established in LIC. These include the Masquelet technique for bone defect reconstruction [22, 23] also common in HIC, the Papineau technique for infected non unions [24], and basic local muscle and cutaneous flaps. Improvisation may generate further feasible alternatives. Usually, the strategy used for the solution needs to be simple and the one that fails best. Time-consuming free flap would probably have high failure rates and compromise the ability to treat a high number of patients. Conclusively, we think that special implants cannot be the solution for these injuries but surgical techniques, such as “NGO surgery.”

Only three cases were treated off-label because the implant was not ready. The reason was that the sterilization of the implant was not on time or incomplete. Although in HIC these adverse events are not acceptable, three cases seem comparably few for NGO hospitals in LIC. In our experience, these organizational flaws are inherent to treatment pathways in these conditions. This might be related to the education of medical personnel in LIC [25]. Usually, the only specialized training for the medical staff takes place in the hospital. The role of nursery in an NGO setting in LIC remains unclear [26]. Usually, this lack of implant is discovered during surgery only and the surgeon has no choice but to use whatever implant is ready or to abort the surgery.

### Limitation

The long-term outcome of the fractures described remains unclear. It is uncertain if our approach is favorable instead of doing nothing, or conservative treatment. But this work reflects the situation of NGO hospitals in LIC. Like in other crises, much remains unknown, and particularly the situation where our medical knowledge is confronted with situations where we have no solution is more common in LIC.

Fractures can be treated conservatively or operatively, depending on the indication, the surgeon ´s expertise, and mostly true for LIC, on the resources and the implants available. The treatment will be conservative anyway, when there are no implants at all. For surgeons working in similar circumstances in LIC, the question is not what the indication is, but what implants or resources are available. The next question is how can these available resources be used to provide the best functional outcome for the injury. Textbook rules may not apply and improvisation is a possibility to improve conservative treatment. The off-label use depends on the injury, the implants available, and the skills of the surgeon. In this NGO hospital, K-wires, external fixators, SIGN nails, non-locking plates, and screws were ready to be combined for the treatment of fractures in a comparably well-equipped OT. Because of its high hygiene standards, the outlook for this NGO OT would be the acquisition of locking plates, as they would probably improve the treatment of trauma patients significantly.

## Conclusion

That NGO surgeons have to work with limited resources and inferior implants is not surprising. However, the surgeon must use the implants available, mostly K-wires and external fixators, to treat complex fractures. Using the implants off-label can help the surgeon to treat fractures otherwise left untreated.

## Data Availability

All data gathered including X-rays can be requested from the authors.
